# Role of irisin in physiology and pathology

**DOI:** 10.3389/fendo.2022.962968

**Published:** 2022-09-26

**Authors:** Shiqiang Liu, Fengqi Cui, Kaiting Ning, Zhen Wang, Pengyu Fu, Dongen Wang, Huiyun Xu

**Affiliations:** ^1^ Key Laboratory for Space Bioscience and Biotechnology, School of Life Sciences, Northwestern Polytechnical University, Xi’an, China; ^2^ Xi’an International Medical Center Hospital Affiliated to Northwest University, Xi’an, China; ^3^ Department of Physical Education, Northwestern Polytechnical University, Xi’an, China; ^4^ Research Center of Special Environmental Biomechanics and Medical Engineering, Northwestern Polytechnical University, Xi’an, China

**Keywords:** irisin, beige fat, musculoskeletal homeostasis, cancer, liver, cardiovascular diseases

## Abstract

Irisin, out-membrane part of fibronectin type III domain–containing 5 protein (FNDC5), was activated by Peroxisome proliferator-activated receptor γ (PPARγ) coactivator-1α (PGC-1α) during physical exercise in skeletal muscle tissues. Most studies have reported that the concentration of irisin is highly associated with health status. For instance, the level of irisin is significantly lower in patients with obesity, osteoporosis/fractures, muscle atrophy, Alzheimer’s disease, and cardiovascular diseases (CVDs) but higher in patients with cancer. Irisin can bind to its receptor integrin αV/β5 to induce browning of white fat, maintain glucose stability, keep bone homeostasis, and alleviate cardiac injury. However, it is unclear whether it works by directly binding to its receptors to regulate muscle regeneration, promote neurogenesis, keep liver glucose homeostasis, and inhibit cancer development. Supplementation of recombinant irisin or exercise-activated irisin might be a successful strategy to fight obesity, osteoporosis, muscle atrophy, liver injury, and CVDs in one go. Here, we summarize the publications of FNDC5/irisin from PubMed/Medline, Scopus, and Web of Science until March 2022, and we review the role of FNDC5/irisin in physiology and pathology.

## 1 Introduction

The protein sequence of fibronectin type III domain–containing 5 protein (FNDC5) contains a signal peptide [for endoplasmic reticulum (ER) targeting nascent FNDC5] (1), a hydrophobic transmembrane domain, a fibronectin III domain (the main part of irisin in the extracellular), and a carboxyterminal domain in the cytoplasm. After being N-glycosylated at the two potential sites—Asn36 and Asn81 (mouse) (2) or Asn7 and Asn52 (human) (3) in the ER—and cleaved by disintegrin and metallopeptidase domain (ADAM) family proteins such as ADAM10 (4), irisin is secreted to the blood circulation.

The physiological role of irisin in inducing thermogenic beige fat genesis to control energy metabolism was first described by Spiegelman and his teammates in 2012 in *Nature* (5). After that, irisin has also been found to promote liver glycogen synthesis and inhibit liver gluconeogenesis to maintain glucose homeostasis (6, 7). Later, the function of irisin in nerve system was found in improving cognition, learning, and memory (8). Moreover, irisin also contributes to maintaining musculoskeletal homeostasis by binding with integrin αVβ5 (9, 10). In recent years, researchers have also revealed that irisin reduces the risk of cancers (11) and cardiovascular diseases (CVDs) (12).

In this review, we summarized the up-to-date publications on irisin. We hope that it will help to understand the mechanisms of irisin and provide clues for the clinical application of irisin in diseases.

### 2.1. Role of irisin in inducing white fat browning

The upregulation of FNDC5/irisin under aerobic exercise (5, 8, 13) or cold-induced shivering (14) induces the “browning” of white fat *via* increasing the expression of thermogenic genes such as *Prdm16*, *Dio2*, *cidea*, *Cox-7a*, *PGC-1α*, and *UCP-1* in white fat. The activated beige fat dissipated energy in the form of heat by absorbing the excessive energy substrates (fatty acid or glucose), which improved obesity and type 2 diabetes mellitus (T2DM). On the basis of the single-cell RNA-seq method, Kajimura and his workmates revealed that irisin induced adipocyte progenitor cells (APCs) toward *de novo* beige fat biogenesis and proliferation by activating a complex of CD81 and αVβ1/β5 integrins to phosphorylate Focal adhesion kinase 1 (FAK, Tyr397) signaling (15, 16). Moreover, injection of recombinant irisin (r-irisin; 0.5 mg/g/day) into diet-induced obese (DIO) mice for 14 days resulted in activating thermogenic genes expression and browning of subcutaneous white fat, thereby reducing body weight and improving glucose metabolism (3). *In vitro* studies suggested that irisin induced the expression of *UCP1* and other thermogenic genes in primary inguinal adipocytes and 3T3-L1 preadipocytes through activating p38 mitogen-activated protein kinase (p38 MAPK) and extracellular signal–related kinase 1/2 (ERK1/2) signaling pathways (3). Inhibition of P38 and ERK1/2 expression could block the upregulation of Uncoupling protein 1 (UCP-1) by irisin, which was called “Irisin ERKs the Fat” by Wu et al. (17). In conclusion, irisin can promote the proliferation and differentiation of beige APCs and the browning of mature white fat.

Most studies have shown that irisin expression is significantly lower in individuals with DIO or T2DM than normal individuals (6, 18–20). However, there are also opposite results, for instance, the concentration of irisin in the blood of obese individuals is higher than that of thin individuals (normal) (21–23) under sedentary conditions. A meta-analysis with 1,005 obese patients and 1,242 control subjects showed that obese individuals had a higher circulating irisin level (24). In the development of obesity, the high level of irisin was probably derived from the increased in white adipose tissue (WAT) (25), as the expression of FNDC5/irisin was decreased in muscle tissues (26), as well as in brown adipose tissue and other tissues. However, if irisin concentration is indeed elevated in obese individuals, then why is not irisin doing its job of burning and “ERK-ing” the fat? In obese adipose tissues, is it reduced in the expression of its receptor αVβ1/β5 integrins or the sensitivity? This controversial phenomenon was also assumed as “irisin resistance”. Clearly, these questions require further exploration in the future.

### 2.2. Role of irisin in the liver

The liver is the main site of gluconeogenesis and glycogenesis to maintain energy metabolism. Irisin expression was significantly reduced in subjects with steatohepatitis (6, 18, 19) or in mouse models of ischemia-reperfusion (I/R)–induced liver injury (27, 28). Long-term exercise-induced irisin or supplementation of exogenous r-irisin could protect the liver from non-alcoholic fatty liver disease (NAFLD) (6, 7), liver glucose disorder (29, 30), or I/R-induced liver injury (31), which embodied the potential role of irisin in muscle-liver cross-talk (32).

Hong et al. (33) revealed that injection of r-irisin into DIO mice for 2 weeks inhibited hepatic cholesterol synthesis *via* activating Adenosine 5‘-monophosphate (AMP)-activated protein kinase (AMPK, Thr172) and inhibiting sterol regulatory element-binding transcription factor 2 (SREBP2) expression. Hepatic glucose homeostasis is closely related to hepatic gluconeogenesis and glycogen synthesis. Studies have found that irisin reduces the expression of phosphoenolpyruvate carboxykinase (PEPCK) and glucose-6-phosphatase (G6Pase)–mediated gluconeogenesis in the liver, which can be weakened by suppressing AMPK Small interfering RNA (AMPK siRNA), suggesting that irisin inhibits gluconeogenesis through activating AMPK-PEPCK/G6Pase pathway (30). Similar results also revealed that irisin inhibited glucosamine (GlcN) or palmitate-induced primary hepatocyte insulin resistance by activating phosphatidylinositol 3-kinase (PI3K)/Protein kinase B (Akt)/forkhead box protein O1 (FOXO1)–mediated PEPCK and G6Pase; meanwhile, irisin augments liver glycogenesis through PI3K/Akt/GSK3–glycogen synthase (GS) signaling pathway (29). A recent study has revealed that exercise-induced irisin competitively inhibits the binding of myeloid differentiation factor 2 (MD2) and Toll-like receptor 4 by forming a complex with MD2 in liver cells and thus inhibits the inflammatory response, which may contribute to the improvement of NAFLD by reducing liver steatosis and fibrosis through exercise (34).

I/R is a leading reason of liver injury after liver resection or transplantation (35), which highly associated with liver steatosis (36). During I/R, irisin expression was significantly reduced in serum and liver tissues (27, 31). Intravenous injection of irisin (250 μg/kg) significantly attenuated the I/R-induced decrease of mitochondria, the increment of apoptotic liver cells, high expression of inflammatory factors, and oxidative stress in the liver (27). Studies have found that supplemental irisin can bind to integrin αVβ5 and activate the downstream AMPK-UCP2 pathway to protect intestinal epithelial cells against I/R-induced cell apoptosis and oxidative stress (37).

In conclusion, irisin acts as an anti-obesity and anti-diabetic factor *via* regulating glucose and cholesterol synthesis metabolism in the liver. It enhances liver glycogen synthesis by activating PI3K/Akt/GSK3-GS pathway and inhibits gluconeogenesis *via* activating AMPK-PEPCK/G6Pase as well as PI3K/Akt/FoxO1-PEPCK and G6Pase pathway in the liver. In addition, irisin alleviates inflammation and oxidative stress in liver injury induced by I/R. However, there are still some problems that are not clear enough. The role of irisin receptor in the liver is rarely reported, and whether irisin first activates receptors on the surface of hepatocyte and then regulates glucose and lipid metabolism is still unclear.

### 2.3. Role of irisin in nerves

FNDC5/irisin as a novel therapeutic factor capable of improving cognition, learning, and memory function (38), which has been proved in brain injury caused in cerebral ischemia (39), stroke (40), and anxiety (41). The mediator role of FNDC5/irisin in the brain was first described by Spiegelman and his co-workers in 2013 (8); it was shown that RNA interference (RNAi)-mediated knockdown of FNDC5 reduced brain-derived neurotrophic factor [BDNF; key factor for neuronal cell survival, synaptic plasticity, dendritic arborization, and synaptogenesis (42, 43)]; reversely, increasing irisin levels in the blood by delivery FNDC5 with adenovirus increased the expression of BDNF and other neuroprotective factors, which opened a new avenue in skeletal muscle-brain cross-talk.

Moreover, *fndc5* knockout Knock out (KO) mice had abnormal morphology and function of dentate gyrus neurons (part of the hippocampus), and the cognitive function of the mutant mice was significantly inhibited. Direct or peripheral delivery of irisin to the dentate gyrus was sufficient to ameliorate the cognitive deficits and neuropathology of the mice (44). In the mouse cerebral ischemia model induced by middle cerebral artery occlusion, the level of irisin was negatively correlated with the cerebral infarction volume and brain function injury score, whereas in those treated with r-irisin, the cerebral infarction volume, nerve function injury, and brain edema of the mice were significantly improved, which were related with phosphorylation of ERK1/2-Akt–mediated inflammation (39). Recently, integrin αV/β5 in the hippocampus and cortex was detected (45), which makes it available that irisin combines with integrin αV/β5 to exert the protective effect on brain; however, the specific mechanism remains to be further explored.

In addition, Lourenco et al. showed that the FNDC5/irisin expression was reduced in hippocampi and cerebrospinal fluid of Alzheimer’s disease (AD) models. Inhibition of FNDC5 in the brain impaired the memory system of mice and blocked the neuroprotective effect of physical exercise on synaptic plasticity and memory. Conversely, the increased irisin levels in AD mice improved synaptic plasticity and memory (38). *In vitro*, r-irisin prevented neurons from Aβ- (25–35)–induced cell toxicity *via* attenuating IL-6– and IL-1β–mediated inflammation status (46). Furthermore, irisin was the upstream regulator of BDNF, which attenuated the learning and memory deficits as well as the cytotoxic response against Aβ toxicity in AD (47, 48). Thus, the activation of the irisin–BDNF axis may be a potential therapeutic target for AD (49).

In conclusion, the expression of irisin was decreased in patients with brain injury. Exogenous r-irisin supplementation significantly protects nerves and enhances memory and cognitive function. Thus, irisin can be used as a potential target for the treatment of stroke, cerebral ischemia, AD, and other brain injuries.

### 2.4. Role of irisin in bone

#### 2.4.1. Effects of irisin on bone tissues

Musculoskeletal interaction is one of research hot spots in recent years (50, 51). Colaianni et al. (52) found that conditioned medium (CM) from primary myoblasts of mice after 3 weeks of exercise induced a higher degree of osteoblast differentiation *in vitro* than that under resting conditions, and adding neutralizing antibody of FNDC5/irisin into the CM significantly reduced the expression of alkaline phosphatase (ALP) and collagen I (Col I) in osteoblasts. That was the first to establish that irisin secreted from muscles has a positive regulatory effect on bone during physical exercise. After that, Zhang et al. (53) found that 2 weeks of free wheel-running exercise increased the expression of osteogenic markers such as osterix (Osx), bone sialoprotein (BSP), and osteocalcin (OCN), as well as FNDC5/irisin in bone tissues. Irisin receptor integrin αV mediated the modulation of irisin on bone during exercise. Eight weeks of running exercise–inhibited ovariectomized (OVX) induced the reduction of femoral trabecular and cortical bone mineral density (BMD) (54), and exercise improved bone microarchitecture and increased the number of ALP-positive cells in OVX mice, whereas twice-weekly injection of cyclo RGDyk polypeptide drugs (anti-irisin receptor integrin αV agents) weakened the improvement effects of exercise (55).

The concentration of FNDC5/irisin was strongly correlated with BMD and bone homeostasis. A cross-sectional and case-control study showed that low concentrations of irisin in serum were related to hip fractures and osteoporosis in postmenopausal women (56, 57). FNDC5/irisin deletion in osteoblast lineage resulted in a lower bone density and delayed bone development and mineralization in mice, FNDC5/irisin KO also blocked the increment of cortical bone thickness by 4 days of voluntary wheel-running exercise (58). Systemic FNDC5 KO mice resulted in low bone strength and mass than Wild type (WT) mice (59), and global FNDC5/irisin KO also completely blocked OVX-induced osteocytic osteolysis and trabecular bone loss (60).

#### 2.4.2 Role of irisin in osteoblasts

Bone modeling and remodeling require the balance of osteoblasts-induced bone formation and osteoclasts-induced bone resorption (61). Irisin activates osteogenic gene expression and induces bone formation. Injecting r-irisin (100 μg/kg/weeks, 4 weeks) significantly increased the mRNA expression of activating transcription factor 4 (*Atf4*) in bone marrow and phosphoprotein 1 (osteopontin, Spp1) in the whole tibia, indicating that irisin shifted from mesenchymal stem cell commitment toward osteoblast lineage and increased bone formation; *in vitro* experiments showed that r-irisin upregulated osteoblast marker genes like *Bmp2/4*, *Spp1*, Runt-related transcription factor-2 (*Runx2*), *Alp*, and *Atf4*, as well as phosphorylation of ERK1/2 in bone marrow stromal cells (62).

Furthermore, administration of r-irisin (100 ng/ml) induced differentiation and mineralization of primary rat osteoblasts and MC3T3-E1 cells by increasing the expression of osteoblast transcription regulators and differentiation marker, which was blocked by inhibiting p38 and ERK1/2 expression (63). Physical exercise activated Akt-β-catenin (essential for osteoblastic differentiation (64)) and induced ALP-positive cells increment, and these effects were abolished by tail vein injecting integrin αV inhibitor (55), which suggested that irisin increased bone mass by binding to osteoblast surface receptors and activating the Akt/β-catenin-Alp pathway. Recently, Xue et al. (65). also got a similar result in preosteoblasts.

#### 2.4.3. Role of irisin in osteoclasts

Irisin protects bone microstructure by stimulating osteoblasts production and inhibiting the differentiation of osteoclasts to establish a “new balance”. Injection of r-irisin into OVX-induced mice significantly increased the number of osteoblasts on the surface of trabeculae bone while inhibiting the number of osteoclasts and decreasing the concentration of tartrate-resistant acid phosphatase (TRAP; marker of osteoclasts) (66, 67).

In addition, supplementation of r-irisin (20 nmol/L) in pre-osteoclastic RAW264.7 cells for 4 days resulted in the decrease of osteoclast differentiation markers (53). Moreover, Ma et al. (68) showed that irisin promoted the proliferation of two osteoclast precursor cells (RAW264.7 cells and mouse bone marrow monocytes) *via* activating p38-MAPK and c-Jun N-terminal kinase (JNK) signaling pathways but significantly downregulated osteoclasts differentiation markers, as well as decreased hydroxyapatite resorption pits and TRAP^+^ multinucleated cell numbers. However, there were also some different results. Estell et al. (69). found that administration of irisin (2–10 ng/ml) promoted the differentiation of mouse bone marrow progenitors toward osteoclasts and that overexpression of fndc5 in mice promoted the differentiation and resorption of osteoclasts, which resulted in lower bone mass.

#### 2.4.4. Role of irisin in osteocytes

Osteocytes accounted for more than 90% of bone cells and played crucial roles in bone homeostasis. Irisin prevents bone loss and osteoporosis by robustly inhibiting osteocytic apoptosis. Spiegelman and his workmates revealed for the first time that irisin bound directly to osteocytes by integrin receptors (αVβ1/β5) and that inhibition of integrin αV receptor expression significantly inhibited the activation of SOST in bone cells by r-irisin. Injection of r-irisin (100 μg/kg) *in vivo* improved disuse-induced low viability and apoptosis of osteocytes and a high rate of empty lacunae (70). Furthermore, they found that irisin rapidly activated the expression of *Atf4* and inhibited apoptosis by activating ERK1/2 in MLO-Y4 osteocytes, which contributed to bone development (70, 71).

In summary, irisin regulates bone regeneration and homeostasis, which reflects the key regulatory role of muscle on bone (72). We summarized the effects of irisin in bone tissue cells in **Table 1**.

**Table 1:** Role of irisin in bone.

Type cell/Animal	Irisin concentration/Endurance	Main effect	Reference
Mice	100 μg/kg/week; 4 weeks	Atf4**↑,** spp1**↑,** bone formation**↑**	(62)
Primary osteoblast and MC3T3-E1 cell	100 ng/ml; 3 and 14 days	Runx2**↑**, Osx**↑**, ALP**↑**, ColIa1**↑**, p-P38**↑**, p-ERK1/2**↑**	(63)
Murine BMSCs	40 μM; 2, 7, 14, and 21days	Runx2**↑**, OCN**↑**, ALP**↑**, Atg5**↑**, β-catenin**↑**, Lef1**↑**, Tcf4**↑**	(73)
Mouse preosteoblast-like cells MC3T3-E1	100n g/ml; 1, 5, 10, and 20 min; 3, 8, and 24 h; 6 days	P21↓	(74)
Mice	100 µg/kg/week; 4 weeks	ALP**↑**, Col I**↑**, BMD**↑**	(10)
Primary murine OC, MC3T3E1	100 ng/ml; 14 days	Runx2**↑**, Atf4**↑**, Osterix**↑**, Col I**↑**, Osteoprotegerin**↑**, Trap (×), Cathepsin K (×)	(75)
Primary osteoblasts, MC3T3-E1	1 nM; 24, 48, and 72 h; 14 days	ColIa1**↑**, ALP**↑**, calcium deposition**↑**, β-catenin**↑,**	(76)
RAW264.7 cells	20 nmol/L; 4 days	NFATc1↓, CK ↓, Trap↓	(53)
RAW264.7 cells, mouse bone marrow monocytes	20 and 40 nM; 4 and 5 days	RANK↓, CK ↓, Trap↓ differentiation↓	(68)
MLO-Y4	100 ng/ml; 1, 5, 10, 20, and 60 min; 6 days	p-ERK1/2**↑**, Atf4**↑**, SOST↓, caspase3/9↓	(70)
Mice	18 ng/ml; 3× a week for 4 weeks	TNF-α↓, IL-17↓	(77)

↑: Increased, ↓: Decreased, ×: No change

### 2.5. Role of irisin in skeletal muscle

AMPK–PGC-1α (PPARγ coactivator-1α)–FNDC5 axis is the most important pathway for irisin synthesis. During exercise, the Ca^2+^ level is increased significantly in the muscle cytoplasm along with skeletal muscle contraction and then stimulates the phosphorylation of AMPK (78), which, in turn, enhances PGC-1α and regulates transcription of downstream factors such as *fndc5*. Shan et al. revealed that myostatin (*MSTN*) KO in skeletal muscle significantly increased PGC-1α and FNDC5/irisin expression, and the high level of irisin increased the browning of WAT in MSTN^−/−^ mice (79). Moreover, Ge et al. found that myostatin inhibited FNDC5/irisin expression by increasing miR-34a (80).

The blood circulation level of irisin has been identified as a biomarker for muscle mass and performance (81). For example, the concentration of irisin in patients with sarcopenia and pre-sarcopenia was lower compared with that in non-sarcopenic participants (82, 83). Exposure to an ambient hypoxic environment can cause skeletal muscle loss and atrophy, along with the low concentration of irisin in blood circulation both in humans (84) and mice (85), which could be one of the reasons of muscle atrophy induced by hypoxia (86). However, interestingly, knockdown of fndc5 in skeletal muscle still performed equal muscle mass, development, growth, regeneration, and strength compared with WT mice. Although, there was no difference in cardiotoxin-induced muscle injury between fndc5-mutant and WT mice (87).

Multiple studies showed that exogenous r-irisin improved skeletal muscle loss and atrophy. Colaianni et al. revealed that r-irisin prevented hindlimb unloading-induced muscle mass decline and decrease of myosin type II expression (10). In addition, *in vitro*, *fndc5* gene expression and irisin concentration were positively correlated with the process of differentiation of C2C12 myotubes; r-irisin supplementation increased human primary skeletal muscle cell growth and hypertrophy by increasing insulin-like growth factor 1(IGF-1)/PGC1α4 and decreasing myostatin through activating ERK1/2 pathway (88). Another study from Reza et al. showed that r-irisin increased myogenic differentiation and myoblast fusion *via* activating IL-6 signaling pathway, and r-irisin treatment also improved denervation-induced muscle injury by increasing protein synthesis through the ERK1/2 pathway (9). Irisin treatment (100 ng/ml, 24 h) also prevented dexamethasone-induced atrophy in C2C12 myotubes by upregulating IGF-1 and attenuating proteolytic activity through dephosphorylation of FoxO3α-mediated ubiquitin-proteasome overactivity (89).

In short, irisin is mainly produced by muscle tissue *via* Ca^2+^–AMPK–PGC-1α–FNDC5 pathway. It induces the expression of myoblasts by activating downstream ERK1/2 and IL-6 pathways in an autocrine manner, which plays key regulatory role in muscle growth and differentiation. However, there are still some problems, such as whether the receptor is still integrin αV/β5 on the surface of muscle cells. In addition, the role of integrin αV/β5 in exercise-induced muscle hyperplasia and hypertrophy is unclear.

### 2.6. Role of irisin in articular cartilage

Osteoarthritis (OA) is a degenerative joint injury characterized by joint pain, progressive cartilaginous degeneration, and stiffness, which poses a great challenge to the physical health of the patients (90). It was stated that FNDC5/irisin activated by moderate physical exercise played a key role in alleviating symptoms and the process of OA such as progressive cartilaginous degeneration, synovial inflammation, and osteophyte formation (71, 91, 92). Studies have shown that the expression of FNDC5/irisin is reduced in patients with osteoarthritic cartilage (93) or synovial fluid (94) compared with that in healthy subjects. In addition, FNDC5 KO mice accelerated anterior cruciate ligament transection–induced OA progression; conversely, FNDC5 knock-in attenuated OA progression (95).

Direct intraarticular injection of irisin may be more effective as almost no blood vessels pass through cartilage. Destabilized medial meniscus (DMM)–induced OA mice were directly injected intra-articular with r-irisin for 8 weeks; the results showed that irisin prevented articular cartilage loss and ameliorated irregular gait; moreover, administration of irisin increased autophagy flux and survival of chondrocytes in DMM-induced OA mice by increasing the expression of LC3 and proliferating cell nuclear antigen (93). *In vitro*, Col II and tissue inhibitor of matrix metalloproteinase (MMP)–1 and –3 expression was significantly increased, and Col X (hypertrophic chondrocyte–related gene) and MMP-1 and MMP-13 expression significantly decreased by adding r-irisin to human primary chondrocytes for 7 days, indicating that the addition of irisin contributes to maintaining partial stability of the extracellular matrix (ECM) of cartilage (96). This mechanism may be related to irisin, lowering the activation of p38, JNK, and Akt in chondrocytes (96, 97). Recently, Jia et al. (98). found that exercise-activated irisin alleviated OA chondrocyte inflammation by inhibiting PI3K/Akt/Nuclear factor kappa B (NF-κB) signaling pathway and suppressing the NOD-like receptor protein 3 (NLRP3) and caspase-1–mediated pyroptosis. In addition, *in vitro*, r-irisin treatment (5 and 10 ng/ml, 24 h) attenuated IL-lβ–induced PI3K/Akt/NF-κB p65 cascade and blocked the nuclear translocation of NF-κB p65. Furthermore, irisin supplementation also improved the inflammatory status of OA by reducing the expression of inflammation factors such as IL-1β (95, 99), TNF-α (92), IL-6, and IL-1 (96).

In summary, the expression of irisin was reduced in patients with OA, and moderate physical exercise could alleviate OA by activating irisin (92). The therapeutic effect of irisin is mainly reflected in reducing the inflammatory state of damaged cartilage and increasing the autophagy flux; additionally, intraarticular injection of r-irisin may be effective for rehabilitating patients with OA.

### 2.7. Role of irisin in cancer

Cancer is one of the leading causes of human death. Regular exercise helps reducing the risk of cancer (100); as an exercise gene (101), the role of FNDC5/irisin in the occurrence and prevention of cancer has received extensive attention (102). Most studies have shown an elevated irisin expression in cancer (103–105). However, a few studies also reported that irisin expression is reduced in patients with cancer (106). Therefore, more research studies are needed to explore the role of irisin in cancer.

*In vitro*, r-irisin inhibited the proliferation, migration, invasion, and epithelial-to-mesenchymal transition (EMT) in lung cancer (11), epithelial ovarian cancer (107), and pancreatic cancer (PC) (108) cells by inhibiting PI3K/Akt- and Signal transducer and activator of transcription 3 (STAT3)-mediated (109) downstream Snail expression (an important role in stimulating EMT).

Irisin induces the arrest of cancer cell division and inhibits cell growth. Huang et al. revealed that irisin induced G (2)/M cell cycle arrest and increased the expression of P21 and tissue factor pathway inhibitor 2, thereby inhibiting the proliferation and invasion of glioblastoma multiforme cells (91). Similarly, Liu et al. found that the receptor of irisin also existed on the surface of PC cells; supplementation of both non-glycosylated and glycosylated r-irisin in PCs could induce G1 arrest and inhibit the growth of PC *via* activating AMPK and inhibiting mTOR expression (110); these results indicate that irisin can affect tumor tissues and exert antitumor properties. However, there is still not enough evidence that irisin can directly act on the integrins on tumor cells to inhibit the development of EMT or tumor proliferation (111).

Overall, irisin has a wide application prospect for the treatment of cancer. Irisin inhibited the proliferation, migration, and invasion of tumor cells by inhibiting PI3K/Akt- and STAT3-mediated Snail/EMT pathways. In addition, irisin also inhibited tumor growth by inducing G1 or G (2)/M cell cycle arrest through AMPK/mTOR pathway.

### 2.8. Role of irisin in myocardium and blood vessel

CVDs include hypertension, coronary artery disease, myocardial infarction, heart failure, atherosclerosis, and myocardial I/R injury, which are the leading cause of human death worldwide (112). Regular exercise can reduce the risk of CVDs, and irisin may play a crucial role in it. Studies have found that the expression of irisin in patients with CVDs is significantly lower than that in healthy people (113–117). Li et al. revealed that resistance exercise could activate the release of irisin from skeletal muscle and then stimulate the AMPK-PINK1/Parkin-LC3/P62 signaling pathway, which regulated mitophagy and inhibited oxidative stress in the myocardium (12). *In vitro*, studies have shown that irisin binds directly to the endothelial cell surface receptor integrin αV/β5, thereby phosphorylating AMPK (Thr172) and activating PGC-1α (induce mitochondrial biogenesis) and mitochondrial transcription factor A (a key activator of mitochondrial transcription and a participant in mitochondrial genome replication).

Cardiac hypertrophy progresses to heart failure; irisin can significantly improve myocardial hypertrophy. Qing et al. showed that administration of r-irisin could attenuate angiotensin II (Ang II)–induced cardiomyocyte hypertrophy, *in vitro*, and that treatment of irisin in transverse aortic constriction (TAC)–induced cardiac hypertrophy murine, *in vivo*, significantly suppressed cardiac hypertrophy and fibrosis by phosphorylating AMPK (Thr172) and inhibiting the phosphorylation of mTOR (Ser2448). However, the expression of irisin increased in the hypertrophic heart and serum during this period, which may be a stress response from the body, as the elevated irisin could decrease endothelial damage by suppressing oxidative stress and inflammation (4, 118). Yue et al. found that r-irisin protected myocardial hypertrophic mice induced by TAC or Ang II–treated cardiomyocytes *via* inhibiting NLRP3-mediated pyroptosis (119).

The therapeutic role of irisin on cardiac hypertrophy was also reflected in the improvement of autophagy flux and induction of protective autophagy. Li et al. found that supplementation of irisin in Ang II–treated cardiomyocytes significantly increased the expression of LC3II and decreased P62 expression and activated the phosphorylation of AMPK (Thr172) and ULK1 (Ser555), thereby reducing cardiomyocyte apoptosis, and this protection will be reversed by autophagy inhibitor such as 3-methyladenine, autophagy-related 5 siRNA (ATG5), and chloroquine; moreover, blockage of AMPK and ULK1 also abrogated autophagy flux and indicted irisin-induced protective autophagy in cardiac hypertrophy *via* activating AMPK-ULK1 pathway (120, 121).

Growing evidence suggests that the content of irisin in patients with atherosclerosis is significantly lower than that in normal controls (122–124), and irisin supplementation has a significant effect on the treatment and improvement of atherosclerosis. For example, irisin supplementation can significantly improve endothelial dysfunction, decrease endothelial apoptosis, and predominantly decrease atherosclerotic plaque area in nicotine or streptozotocin-induced apolipoprotein E-Null [apoE(^−/−^)] atherosclerosis mice (125). Here, we enumerated the role of irisin in atherosclerosis disease in **Table 2**.

**Table 2:** The role of irisin in atherosclerosis.

Type cell/Animal	Irisin concentration/Endurance	Main effect	Reference
APOE^−/−^ mice	0.02 μg/μl, 2× a week for 3 weeks	Irisin reversed intimal thickening *via* integrin αVβ5 receptor.	(125)
		Irisin inhibited atherosclerosis progression *via* the integrin αVβ5/PI3K/P27 pathway.	(126)
C57BL/6, human umbilical vein endothelial cells	20 nM for 7 days in mice, 24 h in EC	Irisin increased EC viability, migration, and tube formation *via* Akt/mTOR/Nrf2 pathway.	(127)
ApoE^−/−^ mice	0.02 μg/μl, 2× a week for 4 weeks	Irisin decreased endothelial apoptosis, and predominantly decreased atherosclerotic plaque area.	(128)
RAW264.7 macrophages	20, 40, and 80 ng/ml for 30 min	Irisin reduced lipid accumulation in macrophages and inhibited apoptosis	(129)
Human umbilical vein endothelial cells	0.01, 0.1, and 1 μg/ml for 48 h	Irisin ameliorated inflammation and endothelial dysfunction by inhibiting ROS-NLRP3.	(130)

Overall, the integrin αVβ5 on the endothelial cell surface could be activated by FNDC5/irisin. As a key energy sensor to maintain energy balance and mitochondrial hemostasis (131), AMPK mediated the effect of FNDC5/irisin on mitophagy, oxidative stress, and mitochondrial biogenesis, thereby improving myocardial hypertrophy, myocardial infarction, atherosclerosis, and other cardiac diseases, which reflecting the protection of regular exercise on cardiac health.

## 2 Conclusions

**Figure 1:**
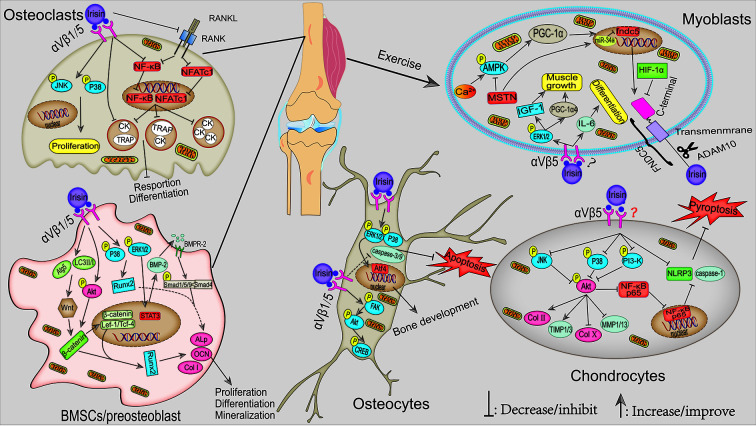
Potential mechanisms signaling pathways for the actions of irisin in musculoskeletal. During exercise, the elevated Ca^2+^ in muscle cytoplasm-induced activation of the AMPK–PGC-1α–FNDC5 axis is the main pathway for irisin synthesis. In addition, irisin, in turn, can stimulate muscle growth and myoblast differentiation *via* ERK1/2–IGF-1/MSTN and IL-6 signaling pathways, respectively. Multiple pathways mediated exercise-induced irisin and r-irisin–activated osteoblast differentiation and mineralization, e.g., p38/ERK1/2, Akt-β-catenin, and Wnt-β-catenin–mediated activation of ALP/OCN/Col I pathways. In osteoclast, irisin induced its proliferation through activating the p38/JNK pathway. In addition, irisin also inhibited the NF-κB and NFATc1 levels in the nucleus, thus inhibiting the expression of osteoclast differentiation marker genes. As for osteocytes, irisin inhibited osteocyte apoptosis by inhibiting caspase-9 and caspase-3 expression, which probably through activating p38/ERK1/2. Furthermore, moderate exercise-activated irisin or r-irisin could alleviate OA by maintaining ECM stabilization and reducing inflammatory response through p38/JNK-Akt and PI3K/Akt/NF-κB signaling pathway, respectively.

**Figure 2:**
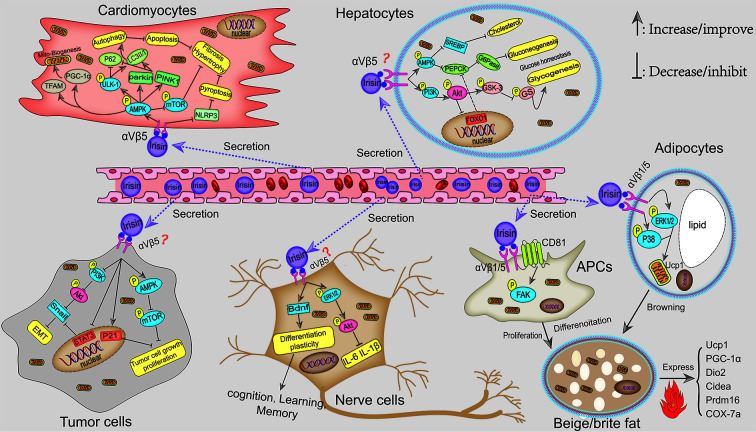
Potential mechanisms signaling pathways for the actions of irisin in the pathological tissues. Irisin protects against DIO by inducing the recruitment of beige fat to dissipate energy into heat. This mechanism is involved in p38 MAPK and ERK1/2 pathways, as well as FAK-mediated beige APCs proliferation. In addition, irisin attenuated diet-induced metabolic disorders, including NAFLD and hepatic steatosis by promoting the synthesis of liver glycogen *via* PI3K/Akt/GSK3-GS and inhibiting the generation of liver gluconeogenesis through AMPK-PEPCK/G6Pase and PI3K/Akt/FOXO1-mediated PEPCK/G6Pase pathways. In brain tissues, irisin promoted cognition and neuro development *via* inhibiting the inflammatory response and activating BDNF-mediated nerve cell survival, differentiation, and plasticity. Moreover, irisin affects the proliferation, migration, and invasion of tumor cells probably by binding integrin αV/β5–mediated PI3K/Akt-Snail-EMT and AMPK-mTOR pathways, which has great therapeutic prospects for inhibiting cancer development. Moreover, exercise-induced irisin can also reduce the risk of cardiovascular diseases. In cardiomyocytes, irisin stimulated AMPK-mediated autophagy and mitobiogenesis by binding to its receptor integrin αV/β5, thereby relieving cardiac hypertrophy and injury.

In this review, we systematically summarized the roles of FNDC5/irisin in fat, liver, nerve, bone, skeletal muscle, articular cartilage, cancer, and angiocarpy. Irisin, as a muscle factor secreted by exercise, plays an extremely important role in regulating fat browning, improving liver and systemic glucose metabolism, maintaining musculoskeletal homeostasis, promoting synaptic growth, and inhibiting the progression of cancer. The mechanism of irisin is mainly through first directly binding to its receptor integrin αV/β1/5 and then activating AMPK, FAK, and MAPK signaling pathways. Collectively, potential mechanisms and signaling pathways for the actions of irisin in musculoskeletal and pathological tissues are shown in **Figures 1** and **2**, respectively.

There are still some unsolved questions, for example, the concentration of irisin in pathophysiological conditions that are highly controversial; some studies suggest that the irisin level rises in patients with obesity or cancer, but why does irisin not play a role in burning and “ERK-ing” the fat as well as inhibiting the development of cancer? Perhaps, its receptor sensitivity and number are reduced under these pathological conditions, which resulted in “irisin resistance”. At this point, high concentration of irisin may not come from muscle tissue but from newly increased fat or cancer tissues; perhaps, due to the decreased activity and expression of its receptor, irisin could not play a substantial role even if the concentration increased. In addition, whether irisin that directly binds to receptors on the surface of chondrocytes, myoblasts, cancer cells, and hepatocytes plays a regulatory role is still unclear, and relevant studies are limited. Therefore, it may be necessary to further explore the role of irisin by detecting the expression of its receptor integrin αV/β1/5 in these pathological and physiological tissues.

Here, we summarized the progress and mechanism of FNDC5/irisin in physiological and pathological conditions, and we analyzed the shortcomings of current research of FNDC5/irisin. We hope that this review may provide an available reference for FNDC5/irisin research.

## Author contributions

SL and FC were mainly involved in data analysis and manuscript drafting, KN and DW helped to draw the mechanic images. ZW and PF made final checks on the manuscript and data. HX provided major theoretical knowledge and relevant suggestions. All authors contributed to the article and approved the submitted version.

## Funding

This work was supported by the National Natural Science Foundation of China (grant numbers 81772409 and 32001055) and the Innovation Foundation for Doctor Dissertation of Northwestern Polytechnical University (grant/award number CX2021098).

## Acknowledgments

Many thanks to Hassan Siddique (University of Science and Technology of China) for checking the grammar.

## Conflict of interest

The authors declare that the research was conducted in the absence of any commercial or financial relationships that could be construed as a potential conflict of interest.

## Publisher’s note

All claims expressed in this article are solely those of the authors and do not necessarily represent those of their affiliated organizations, or those of the publisher, the editors and the reviewers. Any product that may be evaluated in this article, or claim that may be made by its manufacturer, is not guaranteed or endorsed by the publisher.

## Abbreviations

UCP-1, uncoupling protein 1; Dio2, type II iodothyronine deiodinase 2; Cidea, cell death activator; Prdm16, PR domain-containing 16; Cox-7a, cytochrome c oxidase subunit 7a; PINK1, PTEN-induced putative kinase 1; RANKL, receptor activator for nuclear factor–κB ligand; RANK, receptor activator of nuclear factor–κB; NFATc1, nuclear factor of activated T cell 1; TRAP, tartrate-resistant acid phosphatase; CK, cathepsin K; IGF1, insulin-like growth factor 1; FOXO1, forkhead box protein O1; SOST, Sclerostin.
